# Association between polygenic risk for Alzheimer’s disease, brain structure and cognitive abilities in UK Biobank

**DOI:** 10.1038/s41386-021-01190-4

**Published:** 2021-10-07

**Authors:** Rachana Tank, Joey Ward, Kristin E. Flegal, Daniel J. Smith, Mark E. S. Bailey, Jonathan Cavanagh, Donald M. Lyall

**Affiliations:** 1 Institute of Health and Wellbeing, University of Glasgow, Glasgow, UK; 2 Institute of Neuroscience and Psychology, University of Glasgow, Glasgow, UK; 3 Centre for Clinical Brain Sciences, Division of Psychiatry, University of Edinburgh, Edinburgh, UK; 4 School of Life Sciences, College of Medical, Veterinary and Life Sciences, University of Glasgow, Glasgow, UK; 5 Institute of Infection, Immunity & Inflammation, University of Glasgow, Glasgow, UK

**Keywords:** Risk factors, Predictive markers, Genetic markers

## Abstract

Previous studies testing associations between polygenic risk for late-onset Alzheimer’s disease (LOAD-PGR) and brain magnetic resonance imaging (MRI) measures have been limited by small samples and inconsistent consideration of potential confounders. This study investigates whether higher LOAD-PGR is associated with differences in structural brain imaging and cognitive values in a relatively large sample of non-demented, generally healthy adults (UK Biobank). Summary statistics were used to create PGR scores for *n* = 32,790 participants using LDpred. Outcomes included 12 structural MRI volumes and 6 concurrent cognitive measures. Models were adjusted for age, sex, body mass index, genotyping chip, 8 genetic principal components, lifetime smoking, apolipoprotein (*APOE*) e4 genotype and socioeconomic deprivation. We tested for statistical interactions between *APOE* e4 allele dose and LOAD-PGR vs. all outcomes. In fully adjusted models, LOAD-PGR was associated with worse fluid intelligence (standardised beta [β] = −0.080 per LOAD-PGR standard deviation, *p* = 0.002), matrix completion (β = −0.102, *p* = 0.003), smaller left hippocampal total (β = −0.118, *p* = 0.002) and body (β = −0.069, *p* = 0.002) volumes, but not other hippocampal subdivisions. There were no significant *APOE* x LOAD-PGR score interactions for any outcomes in fully adjusted models. This is the largest study to date investigating LOAD-PGR and non-demented structural brain MRI and cognition phenotypes. LOAD-PGR was associated with smaller hippocampal volumes and aspects of cognitive ability in healthy adults and could supplement *APOE* status in risk stratification of cognitive impairment/LOAD.

## Introduction

Dementias affect ~47.5 million people worldwide, 60–70% of which are cases of Late Onset Alzheimer’s disease (LOAD). Estimates predict 135 million people will live with dementia in 2050 [1]. As a major health problem of the 21st century, understanding prognosis and improving diagnosis for LOAD is necessary. LOAD is, however, a progressive disease with insidious onset and prior to clinical diagnosis and subsequent progression, individuals will already have experienced considerable cognitive deficits and attendant brain pathology [2]. The strong association of LOAD with age is partly due to an increase in pathological processes and a cumulative effect of risk factors over the lifespan, including the interaction of environmental, genetic and other biological factors. Vascular problems are the most common comorbidity alongside LOAD; however, some research suggests this apparent connection between brain health and heart health may be due to confounding factors revealed by epidemiological studies, such as shared lifestyle factors, cardiometabolic diseases, sex or age [3].

The largest risk factor for LOAD, after age, is the e4 allele of the apolipoprotein E gene (*APOE*) [4]. *APOE e4* (relative to the other alleles - neutral *e3* or potentially protective *e2*) has been associated with increased risk of cardiovascular disease and Alzheimer’s disease (AD), typically playing a role in transporting lipoproteins and cholesterols in addition to being involved in the metabolism of Aβ [4]. The presence of the *APOE* e4 allele, however, is not necessary nor sufficient for LOAD to develop and recent heritability estimates range from 50% to 79% with both common and rare genetic variants contributing [5]. It is not yet clear to what extent individual genetic variants can predict risk and investigating additional genetic risks may reveal more about the pathology and cognitive deficits experienced leading up to LOAD-related cognitive impairment.

LOAD is primarily a neurodegenerative disease and is associated with differences in several brain phenotypes. Structural imaging evaluations using MRI consistently find the entorhinal cortex is affected, with pathological characteristics including hippocampal volume atrophy, smaller brain volumes and widespread loss of cortical thickness [6]. In addition, abnormalities such as brain infarcts or white matter hyperintensities are common features of LOAD. The present study investigated brain structures considered a priori to underlie cognitive ageing and LOAD [7–9].

There have been conflicting reports regarding the utility of LOAD-PGR scores. Mormino et al. [10] studied healthy participants (*N* = 1,322) and found that higher PGR scores were associated with worse memory (*p* = 0.002) and smaller hippocampus (*p* = 0.002) at baseline, as well as with greater longitudinal cognitive decline (memory: *p* = 0.0005, executive function: *p* = 0.01) and clinical progression to LOAD (*p* < 0.0001). Xiao et al. [11] investigated the influence of LOAD risk alleles on structural MRI measures including hippocampal function and cognitive measures (*N* = 231) and found reduced brain function and metabolism in the hippocampus measured by PET and fMRI in healthy individuals who had high PGR scores. However, they found no association of PGR score with cognition measured using a battery of neuropsychological tests. Overall, PGR may be a valuable tool for predicting conversion to LOAD or pathological trajectories however more high-quality, well-controlled data is required, particularly pertaining to possible interaction with other genetic risk factors e.g. *APOE e4* [12, 13].

Previous studies investigating associations of PGR of LOAD and measures of MRI and cognitive functioning have (where applicable) focused on specific aspects of hippocampal function or structure, generally in small sample sizes and without consistently controlling for potential confounding variables like smoking, socioeconomic deprivation or cardiometabolic conditions. The largest previous LOAD-PGR MRI study had a sample size of 3495 [12]. By contrast, our sample size in this study was almost an order of magnitude larger, at *n* = 32,790. We hypothesised that higher genetic loading for LOAD would be associated with worse brain MRI (i.e. smaller volumes; increased lesion burden) and cognitive test scores (e.g. slower processing speed) in generally healthy non-demented adults from the UK Biobank cohort. We also analyse interactions between *APOE e4* dose and PGR score.

## Methods

### UK Biobank dataset

In addition to genetic quality controlling (QC), we also removed participants who reported a neurological condition at baseline (a list of such individuals has been previously published in an open-access report) [14]. This left *n* = 32,790 individuals, aged between 47 and 80 at time of scan. This project was completed using UK Biobank application 17689 (PI: DML).

### Ethical approvals

This study was conducted under generic approval from the NHS National Research Ethics Service (approval letter: 17th June 2011, ref. 11/NW/0382). UK Biobank participants provided written consent at baseline assessment plus later at MRI: http://www.ukbiobank.ac.uk/wp-content/uploads/2011/06/Consent_form.pdf.

### Genotyping

UK Biobank genotyping was conducted by Affymetrix using a bespoke BiLEVE Axiom array for ∼50,000 participants and the remaining ∼450,000 on the Affymetrix UK Biobank Axiom array. Imputation was carried out by UK Biobank based on 1000 genomes phase 3 and UK10k haplotype panels: https://biobank.ndph.ox.ac.uk/showcase/ukb/docs/impute_ukb_v1.pdf. Genetic QC excluded individuals with >10% missing data, genetic sex mismatching self-reported sex, heterozygosity outliers and individuals not of white British ancestry. Genetic QC included single nucleotide polymorphisms (SNPs) with a minor allele frequency greater than 0.01 and SNPs were tested for Hardy-Weinberg equilibrium in both validation and UK Biobank cohorts in which SNPs that had a *p* < 0.001 were not included. This left 6,578,321 SNPs included in the PGR score calculation. Linkage disequilibrium (LD) was calculated using LDpred [15] in 1000 unrelated UK Biobank participants who were not included in final analyses but passed genetic QC, this was to prune the SNP set used for the PGR score for minimal LD.

### Generating polygenic risk scores

PGR scores were generated for all study participants using the infinitesimal model of LDpred [15]. Using LDpred the raw effect sizes are reweighted by linkage disequilibrium (LD) using a reference panel of 1000 unrelated UK Biobank participants. The summary statistics used to create the PGR scores come from an unrelated GWAS meta-analysis using 46 total datasets [5]. A total of 6,578,321 SNPs (including imputed SNPs at a minimum 80% confidence) with varying effect estimates associated with LOAD (onset age >65 years) were included to calculate weighted scores. Polygenic risk scores were standardised to *Z* scores, i.e. mean = 0, standard deviation (SD) = 1 where higher scores equate to increased risk. The two SNPs used to define *APOE* genotype, rs429358 and rs7412, were not included in the set used to calculate polygenic risk scores (i.e. LOAD-PGR thus does not include *APOE* e4 genotype). *APOE e4* allele dose was included as a separate variable for each individual, coded linearly as 0, 1 or 2.

### Brain MRI phenotypes

Twelve MRI volumes (*n* = 32,790) considered to have a priori evidence as major substrates of cognitive decline or reported to be affected in AD were included as outcome measures in this study. These included: total grey matter, white matter, white matter hyperintensity (WMH), whole brain, left hippocampus, right hippocampus [6]; in addition, segmented regions of each left and right hippocampus (head, tail and body) were examined. MRI variables were adjusted for skull size and converted to *Z* scores for interpretation and comparison. WMH was log-transformed due to skewed distribution. All brain MRI data were acquired on a 3T Siemens scanner for which tissue volumes were derived by UK Biobank and available as image-derived phenotypes. Further details about documentation and protocol can be found at: https://biobank.ctsu.ox.ac.uk/crystal/crystal/docs/brain_mri.pdf and https://www.fmrib.ox.ac.uk/ukbiobank/protocol/V4_23092014.pdf.

### Cognitive phenotypes

Cognitive measures used in analyses included: fluid intelligence (i.e. verbal-numeric reasoning) (*n* = 34,509), matrix completion (*n* = 19,310), numerical memory (*n* = 18,397), reaction time (*n* = 34,968), total trail making (A + B) (*n* = 35,138), and symbol digit substitution (*n* = 19,291). Pairs matching and prospective memory were not included in this study due to previously demonstrated poor test-retest reliability; all tests and their methodologies are described in prior open-access papers [14, 16, 17].

### Covariates

Townsend deprivation indices were derived from postcode of residence [18]. This provides an area-based measure of socioeconomic deprivation derived from aggregated data on car ownership, household overcrowding, owner occupation and unemployment. Lower scores indicate more social deprivation. Smoking was coded as number of packs smoked per year as a proportion to lifetime exposure calculated as: pack years/(age at recruitment − 16). BMI was derived from weight (kg)/[height (m)^2^] by UK Biobank. Participants removed their shoes and heavy outer clothing before weight and height were measured. We elected to use 8 genetic principal components as there is evidence that the majority of the variance is explained by 5 GPCs [19] and use of 8 is a common approach.

### Association analyses between PGR, brain MRI and cognition metrics in UK Biobank

Associations between LOAD-PGR score and phenotypes were examined in 32,790 individuals. Standardised LOAD-PGR scores were used as a quantitative variable in association tests (divided into tertiles for reporting of descriptive attributes in Table 1). Regression models for all analyses were partially adjusted then fully adjusted for each MRI and cognitive dependent variable of interest. Partially adjusted models controlled for age at time of MRI scan, genotyping chip and batch, 8 UK Biobank genetic principal components (GPCs) to control for population stratification, sex and BMI. Fully adjusted models were controlled additionally for Townsend deprivation score, smoking (as measured by pack-years as a proportion of lifetime exposure: Number of cigarettes per day/20 * (age stopped smoking − age start smoking)) and APOE e4. Interactions between PGR score and sex, and between PGR score and *APOE e4* dose, were then analysed for each dependent variable to assess if there were differences in LOAD-PGR association magnitude by *APOE e4* allele dose, or alternatively sex, in both partially and fully adjusted models. Standardised betas reflecting differences in SDs, and *p* values for PGR association (themselves scaled to per-SD) are reported. We correct for type-1 error with Bonferroni in which the corrected raw *p* value to reach significance was *p* = 0.003. This was calculated by dividing significance value by number of models run (0.05/18 = 0.003).

**Table 1:** PGR tertiles and demographic statistics.

PGR tertile	*N*	Sex % female	BMI (mean value; SD)	Age (mean years; SD)	Education % degree	Smoking (mean lifetime pack years; SD)	Townsend (mean; SD)	*APOE e4* dose 0/1/2
1	11,152	53%	26.47 (4.32)	63.85 (7.54)	26.20%	0.37 (0.31)	−2.00 (2.1)	73%/24%/3%
2	10,919	52%	26.48 (4.39)	63.76 (7.49)	26.32%	0.39 (0.3)	−1.99 (2.64)	73%/23%/4%
3	10,719	53%	26.5 (4.37)	63.86 (7.43)	25.22%	0.39 (0.3)	−2.03 (2.61)	72%/24%/4%

## Results

Data were available for *N* = 32,790 individuals. Table 1 shows demographic statistics; across the three tertiles, average age was 64 years (SD = 7.5), 52–53% of the sample was female, and 25–26% had a college/university degree.

### Structural volumes

Table 2 shows significant effects were found for left hippocampal volume when models were partially adjusted (standardised β = −0.146 SDs per increased SD of LOAD-PGR, *p* = 0.002, *r*^2^ value = 0.183, i.e. 18% variance explained in outcome explained by this model).

**Table 2:** Associations between polygenic risk for Late-onset Alzheimer’s disease (LOAD-PGR) and MRI volumetric measures.

	Partially adjusted				Fully adjusted			
	Polygenic risk for LOAD		PGR*APOE e4		Polygenic risk LOAD		PGR*APOE e4	
	β	*p*	β	*p*	β	*p*	β	*p*
Left hippocampal volume	−**0.146**	**0.002**	−0.148	0.802	−**0.118**	**0.002**	−0.111	0.363
Right hippocampal volume	−0.128	0.026	−0.116	0.846	−0.071	0.099	−0.234	0.560
Whole brain volume	−0.055	0.201	−0.074	0.439	−0.049	0.075	−0.089	0.615
White matter volume	−0.058	0.351	−0.054	0.911	−0.022	0.115	−0.015	0.138
Log white matter hyperintensity volume	0.026	0.054	**0.034**	**0.002**	0.007	0.549	0.062	0.972
Grey matter volume	−0.173	0.393	−0.017	0.960	−0.051	0.904	−0.074	0.430

β = standardised betas reflecting per standard deviation increased polygenic risk (PGR), and per *APOE* e4 allele. Bold = significant *p* < 0.05. Models are partially adjusted for age, BMI, sex, genotyping chip, 8 genetic principal components and fully adjusted for additional smoking, *APOE e4* dose and social deprivation.

In the fully adjusted model, LOAD-PGR was associated with left hippocampal volume (β = −0.118, *p* = 0.002, *r*^2^ = 0.187); this was the only significant association in this set of models. In terms of interactions, only log WMH showed significant interaction effects of PGR score and *APOE* dose when partially (β = 0.034, *p* = 0.002) but not fully adjusted (*p* = 0.972). There were no significant LOAD-PGR score interaction effects (with *APOE* or sex) for any structural MRI volumes in fully adjusted models.

### Hippocampal subdivisions

Table 3 shows hippocampal subdivisions were associated with LOAD-PGR score. Hippocampal subdivision volumes were lower for left hippocampal head (β = −0.036, *p* = 0.003, *r*^2^ = 0.237) and body (β = −0.056, *p* = 0.002, *r*^2^ = 0.221) when partially adjusted. When fully adjusted, LOAD-PGR was associated with left hippocampal body (β = −0.069, *p* = 0.002, *r*^2^ = 0.119). There were no significant LOAD-PGR score interaction effects (with *APOE* or sex) between PGR and *APOE* for hippocampal subdivision volumes.

**Table 3:** Associations between polygenic risk for Late-onset Alzheimer’s disease (LOAD-PGR) and hippocampal subdivisions.

	Partially adjusted				Fully adjusted			
	Polygenic risk LOAD		PGR*APOE e4		Polygenic risk LOAD		PGR*APOE e4	
	β	*p*	β	*p*	β	*p*	β	*p*
Left Hippocampal head	−**0.036**	**0.003**	−0.030	0.367	−0.014	0.017	−0.013	0.075
Left Hippocampal body	−**0.056**	**0.002**	−0.032	0.167	−**0.069**	**0.002**	−0.051	0.295
Left Hippocampal tail	−0.071	0.167	−0.013	0.075	−0.027	0.016	0.026	0.918
Right Hippocampal head	−0.046	0.023	−0.098	0.679	−0.017	0.044	−0.092	0.563
Right Hippocampal body	−0.029	0.063	−0.023	0.810	−0.031	0.074	−0.041	0.126
Right Hippocampal tail	−0.043	0.935	−0.029	0.809	−0.095	0.932	−0.040	0.113

β = standardised betas reflecting per standard deviation increased polygenic risk (PGR), and per *APOE* e4 allele. Bold = significant *p* < 0.05. Models are partially adjusted for age, BMI, sex, genotyping chip, 8 genetic principal components and fully adjusted for additional smoking, *APOE e4* dose and social deprivation.

### Cognitive abilities

Table 4 shows LOAD-PGR score was significantly associated with five cognitive measures when the model was partially adjusted. These included worse fluid intelligence (β = −0.066, *p* = 5 × 10^−6^, *r*^2^ = 0.082), matrix completion (β = −0.073, *p* = 7 × 10^−5^, *r*^2^ = 0.063), (slower) reaction time (β = 0.022, *p* = 0.003, *r*^2^ = 0.12), (slower) total trail making (β = 0.073, *p* = 2 × 10^−6^, *r*^2^ = 0.079) and worse symbol digit substitution (β = −0.114, *p* = 0.001, *r*^2^ = 0.167). Two measures remained significantly associated with PGR when fully adjusted: fluid intelligence (β = −0.080, *p* = 0.002, *r*^2^ = 0.082) and matrix completion (β = −0.102, *p* = 0.003, *r*^2^ = 0.066). No significant interactions were found for LOAD-PGR score with sex or *APOE* genotype vs. cognitive outcomes (all nominal *P* > 0.05).

**Table 4:** Associations between polygenic risk for Late-onset Alzheimer’s disease (LOAD-PGR) and cognitive function.

	Partially adjusted				Fully adjusted			
	Polygenic risk LOAD		PGR*APOE e4		Polygenic risk LOAD		PGR*APOE e4	
	β	*p*	β	*p*	β	*p*	β	*p*
Fluid intelligence	−**0.066**	**5** **×** **10**^**−6**^	−0.101	0.705	**−0.080**	**0.002**	−0.165	0.769
Matrix completion	−**0.073**	**7** **×** **10**^**−5**^	−0.216	0.499	**−0.102**	**0.003**	0.057	0.393
Numerical memory	−0.232	0.043	−0.011	0.966	−0.039	0.178	−0.054	0.924
Reaction time	**0.022**	**0.003**	0.053	0.701	0.014	0.226	0.048	0.186
Trail making	−**0.073**	**2** **×** **10**^−**6**^	−0.013	0.068	−0.026	0.112	0.027	0.404
Symbol digit	−**0.114**	**0.001**	−0.013	0.876	−0.038	0.090	−0.025	0.885

β = standardised betas reflecting per standard deviation increase in polygenic risk and per *APOE e4* allele increase. Bold = significant *p* < 0.05. Models are partially adjusted for age, BMI, sex, genotyping chip, 8 genetic principal components and fully adjusted for additional smoking, *APOE e4* dose and social deprivation.

### *APOE e4* dose associations

*APOE e4* dose was significantly associated with two MRI measures when partially adjusted: right hippocampal volume (β = −0.039, *p* = 0.002) and white matter hyperintensity (β = −0.042, *p* = 0.002). Right hippocampal volume remained associated when fully adjusted for covariates (β = −0.079, *p* = 0.002). Results showed no significant associations between *APOE e4* dose and cognitive scores for four measures in partially or fully adjusted models (Table 5).

**Table 5:** Associations between *APOE* e4 dose vs. MRI and cognitive measures.

	APOE4 dose partially adjusted		APOE4 dose fully adjusted	
	β	*p*	β	*p*
Whole brain volume	−0.223	0.301	−0.094	0.138
White matter	−0.240	0.347	−0.074	0.206
Grey matter	−0.089	0.430	−0.022	0.302
Left hippocampal volume	−0.064	0.019	−0.069	0.024
Right hippocampal volume	−**0.039**	**0.002**	−**0.079**	**0.002**
White matter hyperintensity volume	**0.042**	**0.002**	0.063	0.006
Left hippocampal body	−0.026	0.065	−0.094	0.048
Right hippocampal body	−0.015	0.347	−0.067	0.046
Right hippocampal tail	−0.026	0.117	−0.076	0.042
Fluid intelligence	−0.064	0.012	−0.069	0.223
Matrix completion	−0.071	0.032	−0.086	0.208
Numeric memory	−0.053	0.085	−0.025	0.648
Reaction time	0.037	0.063	0.042	0.700
Trail making a + b	0.034	0.024	0.059	0.056
Symbol digit	−0.173	0.032	−0.244	0.155

β = standardised betas reflecting per *APOE e4* allele increase. Bold = significant *p* < 0.05. Models are partially adjusted for age, BMI, sex, genotyping chip, 8 genetic principal components and fully adjusted for additional smoking, and social deprivation.

### Sensitivity analysis

In additional analyses, to remove any component of association mediated substantially via frailty, individuals with a BMI <18 were excluded. We did not find that any effect estimates were significantly or meaningfully different with these exclusions. Reaction time and total trail making task A + B estimates were not different when those variables were log transformed.

## Discussion

The current study examined genetic risk of LOAD in 32,790 non-demented adults in UK Biobank and found that LOAD-PGR score was significantly associated with (1) MRI volumes of whole left hippocampus and left hippocampal body, and (2) concurrently assessed cognitive abilities of executive function, namely fluid intelligence and matrix completion, when fully adjusted for covariates including cross-sectional age, BMI, sex, genotyping chip, 8 genetic principal components, smoking, *APOE* e4 genotype and socioeconomic deprivation. Taken together, these results suggest genetic risk variants for LOAD may be able to indicate early signs of pathology prior to significant cognitive problems, and that there is potential for PGR score-based LOAD risk assessment to be made which has relevance for non-demented cognitive abilities and brain health.

### Interpretation

Analyses did not find evidence that LOAD-PGR score was associated with WMH in generally healthy adults. This could be due to vascular risk factors and diseases which tend to be associated with development of WMH, and comorbid with dementias, rather than WMH being linked to dementia directly and in isolation [20]. Armstrong et al. [21] conducted a GWAS for periventricular WMH and deep WMH and found that candidate WMH loci were implicated in stroke, vascular and neuronal functions, but not dementia in isolation. This raises the concern of differentiating LOAD markers from non-LOAD markers such as vascular dementias, which is difficult as AD refers to an aggregate of neuropathological changes assessed post-mortem [2]. It may also be that power to detect such associations is reduced in UK Biobank, as participants have a higher mean vascular health and reduced severity range for vascular conditions compared to the general UK population [22] (an effect heightened due to the exclusion of participants with stents and pacemakers from the cohort recruited for the MRI phenotyping).

The present study found left hippocampus and hippocampal body subdivision showed stronger associations with genetic markers of LOAD, this is consistent with previous studies showing faster left hemisphere atrophy than in right in LOAD [23–26], however, it is important to note that lower volumes in this study may not indicate pathological atrophy or neurodegeneration as only volumetric measures have been taken.

In fully adjusted models, no evidence of interaction between *APOE* e4 genotype and the LOAD-PGR score was found. Any such interaction might have indicated that the main contributions to risk contained within the PGR score operated via mechanisms related to the effect of *APOE* e4 on risk. Genetic interactions are often small in effect and hard to interpret in any case, and it may be that there was limited power in this dataset to see an interaction given the size of each main effect, or there may be inherent limitations in this approach, statistically. Further investigations in larger datasets may be warranted.

### Limitations

The strength of the genetic associations observed in this study may be limited by the methodology of calculating genetic risk scores and modelling assumptions as there is no unified approach to calculating PGR scores efficiently, with variations differently accounting for linkage disequilibrium, beta shrinkage and GWAS *p* value thresholding. It may also be that residual signal coming from *APOE* genotype was present within the LOAD-PGR score, as we did not remove all SNPs in LD with the *APOE* e4-defining SNPs, rs429358 and rs7412, although we did subsequently control for this genotype and therefore to some extent SNPs in LD. In addition, we were not able to replicate genetic effects in an independent cohort. This is due to challenges finding an appropriately phenotyped cohort that was not included within the original GWAS meta-analysis; replication of gene/structural imaging associations is a scientific priority going forward. However, recently published recommendations by Wand et al. [27] were considered when writing this paper, in which reporting standards have been met.

A major limitation of these findings is due to the demographics of the UK Biobank cohort, who are overall less likely to have health conditions, more educated and live in less socioeconomically deprived areas [22]. However, sensitivity analyses showed no results were meaningfully affected by additionally including assessment centre in models. Medication status could also be a limitation as medications that may influence cognitive function such as benzodiazepines or other psychotropics were not considered in this study [28]. Further selection bias may be found for MRI data as data collection is relatively labour intensive for participants and includes contraindications that baseline assessment does not such as stents or pacemakers (which may exclude participants of poorer health). In addition, the findings of this study cannot be generalised to populations outside of White European ancestry [29]. Cohort studies of diverse ancestries are necessary to fully understand prevalence and biological pathways to LOAD.

### Future research

The current analyses use cross-sectional data; future analyses could use longitudinal data to further assess the validity of a PGR score with additional consideration given to *APOE* status, as data are currently conflicting regarding whether e4 vs. e3 influence the rate of progression. It has been hypothesised that e4 genotype may be associated with faster decline due to the association of e4 with additional diseases, creating additive or interactive pathologies. Family history is an informative marker of genetic risk in LOAD, which is often considered in clinical context. It may be useful to test the extent to which that history is an easy proxy for more laborious LOAD-PGR as used here: e.g. a study by Marioni et al. [30] showed that self-reported parental AD was a valid proxy for an AD genetic study.

## Conclusions

This study found evidence that using LOAD-PGR demonstrated significant differences in non-demented brain structure, and to a lesser extent cognitive ability, in generally healthy individuals with a mean age of 64, suggesting PGR may be a useful tool—in combination with other factors—for identifying individuals at risk of worse cognitive abilities and potentially accelerated decline.
